# Training for the Digital Clinical Environment: Preliminary Findings in Implementation of Electronic Health Record Platform in Medical Education

**DOI:** 10.1007/s10916-026-02411-3

**Published:** 2026-06-01

**Authors:** Ashley Tin, Anthony Wong, Kristy Y. Lin, Jack Yang, Avery Buehler, Dongfang Emily Chen, Sounak Dey, Nicholas Stefanko, Christopher Nelson, Aditya Vaidyam

**Affiliations:** 1 Carle Illinois College of Medicine, 506 S Mathews Ave, Urbana, IL 61801 USA; 2 Yale University School of Medicine, 20 York St, CT 06510 New Haven, United States

**Keywords:** Electronic health records, Medical education, Simulation training, Usability, Digital literacy, Clinical documentation

## Abstract

Electronic health records (EHRs) are central to modern clinical workflows, yet undergraduate medical education often provides limited hands-on experience with authentic documentation systems. This gap may reduce preparedness for clerkships and residency, limit confidence with chart navigation, and hinder development of digital literacy and clinical reasoning skills. To develop a learner-centered, open-source, web-based educational EHR platform and evaluate its usability, educational value, and curricular integration across preclinical and clinical learners. A browser-based EHR was developed to simulate real-world documentation workflows, support case-based and problem-based learning, and provide structured feedback. The platform enables instructors to create cases, assign activities, and track learner engagement. Fifty-four preclinical medical students participated in preliminary usability testing and completed post session surveys evaluating usability, learning support, and perceived realism using 5-point Likert scales. Responses were analyzed descriptively. Students reported high usability (mean 4.1 ± 0.6) and strong satisfaction with organization, navigation, and learning support. Learners emphasized workflow realism and integration of documentation with communication tasks. Early EHR exposure helped bridge classroom learning and clinical practice. Feedback informed iterative improvements to interface clarity and workflow design. This platform provides scalable, structured exposure to clinical documentation. Early findings suggest improved digital fluency, confidence, and clinical reasoning. Integration of EHR based simulation into curricula may reduce the learning curve associated with real-world EHR use.

## Introduction

Medical education increasingly emphasizes competency-based and experiential learning, which requires students to develop clinical knowledge along with digital literacy and comfort with tools that shape real patient care. Electronic health records (EHRs) are central to modern clinical workflows, yet many undergraduate medical curricula still provide limited, fragmented, or low-fidelity exposure to these systems. As a result, students often reach the clerkship phase without meaningful practice in documentation, chart navigation, and data synthesis.

National organizations have underscored this gap. The Alliance for Clinical Education (ACE) identifies EHR proficiency as an essential competency for students entering clinical environments and recommends providing structured opportunities for documentation and order entry using realistic platforms [1]. Similarly, the Liaison Committee on Medical Education (LCME), through competencies informed by the Association of American Medical Colleges (AAMC), expects schools to prepare learners to use health information systems effectively [2]. Although most accredited institutions offer some exposure to EHR systems during clinical rotations [3], these experiences vary widely and may not reflect the workflows students will later encounter.

### Current Limitations

Traditional instructional methods such as lectures, paper-based cases, and mannequin-based simulations remain foundational but are not integrated with EHR systems. Many institutions cannot provide access to commercial platforms due to privacy constraints, licensing costs, or operational barriers [4]. Educational alternatives like OpenEMR [5], VistA [6], and locally developed tools often lack the navigation patterns and cognitive demands of dominant commercial platforms. This misalignment is increasingly important because Epic represents over 40% of the EHR market in the United States and is used across more than half of hospital beds nationwide [7]. When training occurs in environments that differ substantially from these systems, learners may develop documentation habits that do not translate effectively to practice.

Prior work also highlights that limited opportunities to document real or simulated encounters restrict skill development and readiness for residency. Welcher et al. [8] argue for longitudinal integration of meaningful, assessable EHR experiences across the undergraduate medical curriculum to support competency-based education.

### Study Objectives

In response to these challenges, we developed a learner-centered, web-based, open-source educational EHR that models authentic documentation workflows and supports case-based and problem-based learning (CBL, PBL) with structured feedback and analytics. To reduce cognitive load and maximize instructional alignment, non-educational components like billing modules were removed. The project’s dual objective is to develop this customizable platform for undergraduate medical education and to evaluate its usability, perceived educational value, and curricular integration via structured learner and faculty feedback. This scalable approach addresses longstanding EHR training deficiencies and aligns with national competency expectations, thereby strengthening digital literacy and clinical reasoning across all training phases.

## Methods

### Platform Architecture

**Fig. 1:**
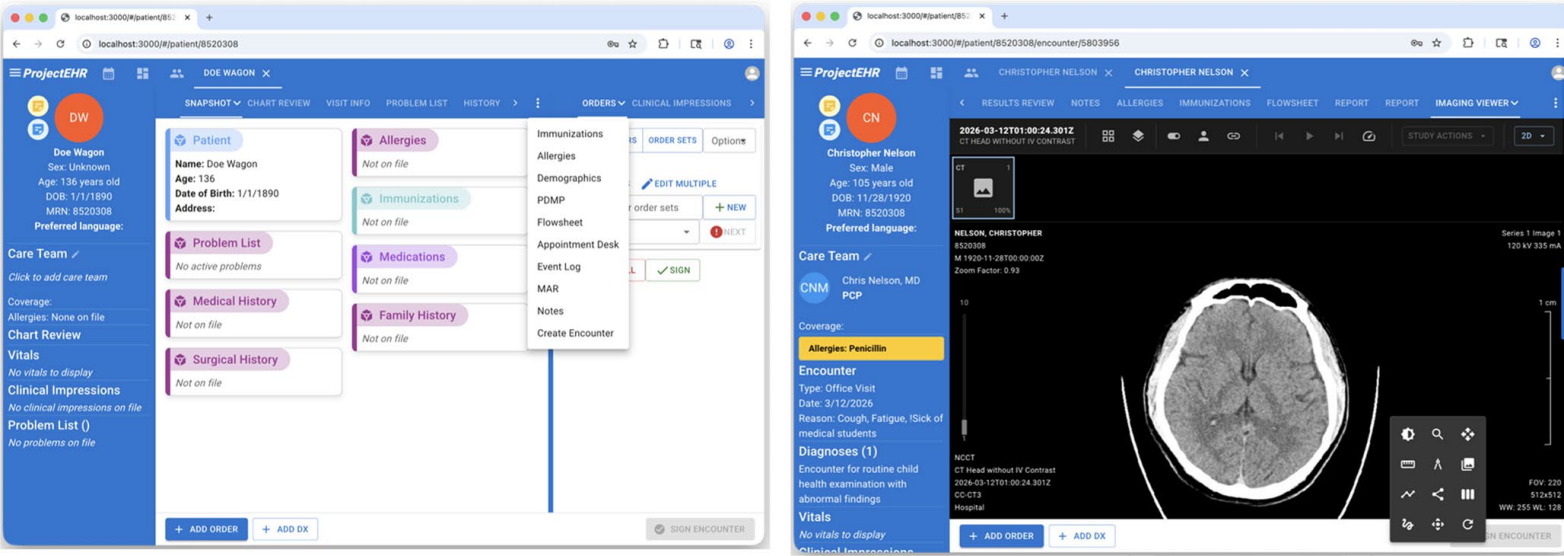
Screenshots of the EHR web application as accessed from the Chrome browser

We engineered a downloadable browser-based EHR to facilitate longitudinal digital clinical exposure. This web application built in ReactJS enables seamless navigation across disparate chart domains, incorporating a DICOM viewer for radiological synthesis and FHIR-aligned data structures to ensure interoperability and didactic scalability. By abstracting complex backend logic, the interface maintains high fidelity while optimizing the user experience. The platform models commercial clinical workflows, facilitating core EHR proficiencies: chart review, documentation, order entry, and diagnostic formulation, including encounters, notes, medications (with reconciliation), labs, imaging, and problem lists (Fig. 1).

The architecture also incorporates an AI-driven Standardized Patient (AI SP) powered by Google Gemini. This multimodal interface facilitates naturalistic history-taking via text or voice, enabling students to practice interpersonal communication with rigorous chart synthesis. This immersive environment cultivates advanced decision-making and clinical acumen without compromising patient safety. Employing FHIR alignment for interoperability and data reuse, the system supports self-directed and instructor-led use across modalities (PBLs, simulations, OSCEs), promoting clinical reasoning and real-time documentation proficiency.

### Development Process

Development of the platform occurred over three years through collaboration among students, educators, clinicians, and software engineers. Iterative prototype testing and monthly faculty review sessions guided decisions related to navigation, workflow alignment, and instructional needs. Design principles were focused on replication of authentic clinical workflows, reduction of cognitive load through removal of non-educational elements such as billing, customizable complexity to support learners at different stages, and integration into active learning environments such as problem-based learning (PBL), simulations, and objective structured clinical examinations (OSCEs).

Medical students across training levels contributed continuously to platform refinement. Feedback from informal testing sessions and short surveys informed improvements to navigation clarity, case organization, and cognitive load. This user-centered process ensured alignment with preclinical learning needs and clerkship preparation. This iterative, stakeholder-driven approach ensured the platform design aligns with both student and faculty needs.

### Study Design

Institutional Review Board (IRB) approval was obtained from the University of Illinois Urbana-Champaign. Seventy-five medical students across different stages of training were recruited from the Carle Illinois College of Medicine for the study, to allow for assessment of the intervention at different points in the curriculum. Participation is voluntary, and students were informed that participation or withdrawal will not affect their academic standing. Written informed consent was obtained from all participants, data was stored in accordance with institutional policies, and only de-identified datasets were used for analysis. Data will be collected using a secure, institution-approved platform that ensures confidentiality and de-identification. Surveys will be administered electronically and linked only to participant ID codes.

A three-arm randomized controlled trial over a longitudinal instructional period is being conducted to evaluate the educational impact of the EHR training platform. To better facilitate development and integration of the EHR, a concurrent usability testing study is being conducted. This usability testing study includes post-session surveys consisting of four questions assessing usability, satisfaction, perceived educational value, and qualitative feedback.

## Results

### Platform Development

The platform successfully replicated core EHR workflows within a simplified, education-focused design. Iterative development resulted in improved navigation, reduced cognitive load, and alignment with authentic clinical workflows. Solutions for technical challenges were developed to support implementation. Limited overhead display availability during simulation-based sessions required a nonintrusive solution to display the EHR without disrupting existing OSCE timers and room interfaces, and thus, a lightweight desktop shortcut was developed to toggle EHR visibility without interrupting simulation workflows. An administrator dashboard was developed based on feedback to allow faculty to assign case visibility to specific stations, supporting structured activities such as OSCEs and enhancing consistency in assessment and instructional design.

### Usability Testing Outcomes

Fifty-four medical students (44 first-years, 10 s-years) participated in initial usability testing (response rate: 84%). Post-session usability survey results are summarized in Table 1. Overall usability ratings were positive (mean 4.1 ± 0.6 on a 5-point scale), with high satisfaction related to interface organization, navigation, and perceived realism. Students agreed that the EHR supported their learning needs and documentation practice.

Qualitative comments described the interface as intuitive and comparable to commercial systems. Preclinical learners noted that early exposure helped bridge the gap between classroom case discussions and clinical workflows. Suggestions included additional orientation materials and small interface refinements, such as improved linking between labs and results. Informal feedback from senior students (third- and fourth-years) indicated that similar tools during their preclinical years would have improved their transition to clerkships. These insights informed subsequent updates to case structure, interface clarity, and feature integration.

**Table 1:** Post-session usability ratings

Survey Item	Mean ± SD	Interpretation
This system meets my information needs.	4.0 ± 0.7	Agree
I am satisfied with this system for learning medical student–specific skills.	4.1 ± 0.6	Agree
Learning to operate this system is easy for me.	4.2 ± 0.7	Agree
I recover easily and quickly from mistakes while using this system.	4.0 ± 0.8	Agree

Scale: 1 = Strongly disagree, 2 = Disagree, 3 = Neutral, 4 = Agree, 5 = Strongly agree

## Discussion

### Principal Findings

This project addresses a persistent and well-recognized gap in medical education. Many students enter clinical training with limited experience navigating electronic health records, synthesizing chart data, or documenting clinical encounters. Traditional approaches such as slide-based cases, printed vignettes, or brief EHR demonstrations provide content knowledge but do not replicate the nonlinear, information-rich workflows encountered in real clinical practice. Our educational EHR is designed to bridge this gap by offering an accessible, realistic, and pedagogically aligned environment that allows learners to practice essential digital competencies early in their training.

Preliminary feedback suggests that simulated chart review supports learner confidence and promotes early familiarity with documentation tasks. Students noted that static simulations obscure the complexity of real EHR workflows, while the platform’s structured notes, interactive prompts, and clinically modeled interface improved navigation and data synthesis. These findings support the need for experiential tools that connect classroom instruction with the digital tasks required in clinical environments.

### Significance and Implications

The platform aligns with emerging competencies that emphasize digital literacy, clinical reasoning, and decision making. By engaging with realistic patient scenarios, learners practice identifying relevant information, generating differential diagnoses, and documenting encounters in a structured, clinically consistent format. The system’s ability to scale complexity by training level, from simplified interfaces for early learners to advanced features for clinical students, also supports competency-based education.

Importantly, the platform is purpose-built for instruction rather than adapted from operational systems. It mirrors the logic and navigation patterns of commercial EHRs but removes administrative features that distract from core learning objectives. This allows students to focus on clinical reasoning and communication while reducing cognitive load.

### Integration Into Medical Curricula

The educational EHR can be incorporated across a wide range of curricular activities. During organ system blocks, students can review charts associated with case-based instruction and order diagnostic tests that reveal sequential information as they reason through cases. This design reinforces the link between clinical decision-making and data generation. The platform can also support objective structured clinical examinations by allowing learners to review charts before simulated encounters, mirroring actual patient care.

Early dry run testing facilitated iterative improvements to visual clarity, system responsiveness, and case organization, demonstrating the value of continuous user-centered refinement. The platform is now positioned for broader curricular adoption and structured evaluation.

### Limitations

Early usability findings came from a small, informal sample and were primarily qualitative. While helpful for guiding design decisions, these results may not be generalizable. The forthcoming trial will address this limitation through larger sample sizes, validated survey measures, and objective performance assessments.

The platform currently uses static case data. Although the system resembles a commercial EHR in workflow and interface, full support for dynamic longitudinal interactions and inpatient workflows remains in development. This limits the range of scenarios that can be simulated. Case creation also requires some technical skill, which may pose a barrier for instructors without software development experience.

Additional planned platform enhancements include collaborative note-taking to support team-based care, embedded clinical decision support tools, and an expanded analytics dashboard for instructors. Technical enhancements will focus on streamlining case authoring, increasing backend scalability, and integrating with simulation management systems.

### Future Work

An ongoing, concurrent three-arm randomized controlled trial (RCT) is assessing the EHR platform’s impact on learner confidence, usability, and documentation performance. Participants are randomized to receive identical case content via (1) EHR-based cases (chart review, data, notes), (2) EHR cases supplemented with an AI-driven virtual standardized patient interaction, or (3) traditional static materials (control). The primary outcome is the change in learner confidence (pre/post surveys). Secondary outcomes include usability and engagement, assessed using validated instruments like the System Usability Scale (SUS). Objective documentation performance is measured through an Objective Structured Clinical Examination (OSCE) scored by blinded faculty using a standardized rubric. Quantitative data analysis will employ linear mixed effects models for survey data and Analysis of Variance (ANOVA) for OSCE scores, utilizing an intention-to-treat framework in R. Qualitative feedback will undergo thematic analysis to further inform platform evaluation.

## Conclusion

This novel, open-source educational EHR platform bridges the pedagogical gap between theoretical instruction and clinical practice by simulating authentic digital workflows. User-centered refinement revealed that preclinical and advanced learners alike require early exposure to documentation systems to foster digital proficiency and reconcile classroom concepts with modern clinical processes. By modeling the cognitive demands of commercial systems while maintaining curricular flexibility, this customizable tool facilitates longitudinal competency-based training, ultimately preparing future physicians for the high-fidelity digital environments of contemporary healthcare.

## Data Availability

No datasets were generated or analysed during the current study.

## Abbreviations

ACE: Alliance for Clinical Education
AAMC: Association of American Medical Colleges
EHR: Electronic Health Record
FHIR: Fast Healthcare Interoperability Resources
AI SP: Artificial Intelligence-based Standardized Patient
LCME: Liaison Committee on Medical Education
OSCE: Objective Structured Clinical Examination
PBL: Problem-Based Learning
RCT: Randomized Controlled Trial
SUS: System Usability Scale
M1: First year medical student
M2: Second year medical student
M3: Third year medical student
M4: Fourth year medical student

## References

[1] Triola MM, Huwendiek S, Levinson AJ, Cook DA, Lowenstein J, Mennin S (2012) New directions in e-learning research in health professions education: report of two symposia. Med Teach 34(1):e15–e20. 10.3109/0142159X.2012.638010 22250691 10.3109/0142159X.2012.638010

[2] Liaison Committee on Medical Education (LCME) (2025) Publications. https://lcme.org/publications/. Accessed 9 Feb 2026.

[3] Association of American Medical Colleges (AAMC) (2023) Webinar: Integrating Electronic Medical Records into Medical Education (Group on Information Resources). https://www.aamc.org/professional-development/affinity-groups/gir/webinar-emrs-meded. Accessed 9 Feb 2026.

[4] Wittels K, Wallenstein J, Patwari R, Patel S (2017) Medical student documentation in the electronic medical record: patterns of use and barriers. West J Emerg Med 18(1):133–136. 10.5811/westjem.2016.10.31294 28116025 10.5811/westjem.2016.10.31294 PMC5226747

[5] Sunchu K, Moncy MM, Purkayastha S, Fulton CR (2025) Lessons learned from OpenEMR implementation in graduate health informatics curriculum. AMIA Annu Symp Proc 2024:1079–1088. 40417551 PMC12099383

[6] Protti D, Groen P (2008) Implementation of the Veterans Health Administration VistA clinical information system around the world. Healthc Q 11(4):83–89. 19068935

[7] Jercich K (2024) Epic gaining more ground: hospital EHR market share widens its lead over Oracle Health. Fierce Healthcare. https://www.fiercehealthcare.com/health-tech/epic-gaining-more-ground-hospital-ehr-market-share-widens-its-lead-over-oracle-health. Accessed 9 Feb 2026.

[8] Hammoud MM, Dalymple JL, Christner JG, Stewart RA, Fisher J, Margo K, Ali II, Briscoe GW, Pangaro LN (2012) Medical student documentation in electronic health records: a collaborative statement from the Alliance for Clinical Education. Teach Learn Med 24(3):257–266. 10.1080/10401334.2012.692284 22775791 10.1080/10401334.2012.692284

